# Active Suppression of Differential Light Shift Drift in an Atom Gravimeter

**DOI:** 10.3390/s26051620

**Published:** 2026-03-04

**Authors:** Wei-Hao Xu, Xi Chen, Jin-Ting Li, Dan-Fang Zhang, Wen-Zhang Wang, Jia-Yi Wei, Jia-Qi Zhong, Biao Tang, Lin Zhou, Run-Bing Li, Jin Wang, Min-Sheng Zhan

**Affiliations:** 1 Innovation Academy for Precision Measurement Science and Technology, Chinese Academy of Sciences, Wuhan 430071, China; xuweihao@apm.ac.cn (W.-H.X.); lijinting@apm.ac.cn (J.-T.L.); zhangdf@apm.ac.cn (D.-F.Z.); wangwz@apm.ac.cn (W.-Z.W.); weijy@apm.ac.cn (J.-Y.W.); jqzhong@apm.ac.cn (J.-Q.Z.); biaotang@apm.ac.cn (B.T.); lzhou@apm.ac.cn (L.Z.); rbli@apm.ac.cn (R.-B.L.); wangjin@apm.ac.cn (J.W.); 2 School of Physical Sciences, University of Chinese Academy of Sciences, Beijing 100049, China; 3 Hefei National Laboratory, Hefei 230088, China; 4 Wuhan Institute of Quantum Technology, Wuhan 430206, China

**Keywords:** atom interferometry, gravity measurement, light shift, systematic error

## Abstract

Differential light shift (DLS) is an important error term that limits the atom interferometer’s measurement precision, especially for the case of the electro-optic modulator (EOM)-based scheme, where multiple laser sidebands exist, and their ratios are hard to control synchronously. This article carried out an experimental and theoretical study on this subject. By conducting long-term gravity measurement, we find that the gravity exhibits drifts of about 13.13 μGal, and is strongly correlated to the Raman laser’s sidebands. A model of the DLS-induced gravity error is established and a DLS compensation method is proposed to suppress the gravity drift to 2.54 μGal. Besides the compensation method, we propose a Dual-Sideband Ratio Locking scheme to more robustly eliminate the gravity measurement drift. By feeding back to both the EOM microwave power and the tapered amplifier’s temperature, this method locks both the ±1 order sideband to a stability level of $10^{-5}$, which corresponds to a gravity error of less than 0.1 μGal. Long-term gravity measurement is carried out after the locking method, showing a long-term stability of 1.6 μGal. The proposed methods will benefit the suppression of the DLS effect for high-precision atom interference measurement.

## 1. Introduction

Atom interferometers use the matter-wave properties of atoms to achieve absolute measurements of inertial quantities through precise coherent manipulation of atomic internal or external states [1]. Due to their high precision and low drift, atom interferometers have become important tools for fundamental physics tests, such as equivalence principles tests [2,3,4,5,6], measurement of the gravitational constant G [7,8], and geophysical applications [9,10,11]. Various techniques have been developed to suppress errors and enhance stability, such as vibration isolation platforms for suppressing vibration noise [12,13,14] and piezoelectric tip-tilt mirrors for compensating phase errors induced by the Coriolis force [15,16].

In the optical architecture of atomic gravimeters, frequency-modulation schemes based on an electro-optic modulator (EOM) are widely adopted due to the fewer optical components and high integration [17,18,19,20]. While pulsed light schemes enhance efficiency [21] and single-sideband (SSB) modulation [22,23,24] provides pure dual frequencies, the EOM associated with the tapered amplifier (TA) architecture remains a common choice for laser power amplification. In this configuration, the Raman light contains three main frequency components: the carrier, the positive, and negative first-order sidebands. It is difficult to independently manipulate the ratio of these three light components using only the EOM radio frequency (RF) driving power as a single degree of freedom.

Laser-atom interactions induce a differential light shift (DLS) via the AC Stark effect [25,26]; therefore, the power ratio of the laser components directly impacts measurement accuracy [27]. In experiments, the “zero light shift point” is usually found by adjusting the intensity ratio via RF power [28,29], combined with the k-vector reversal method [30] to eliminate the error. However, environmental factors such as EOM temperature variations [31] cause random drifts in the sideband ratio. Furthermore, due to potential variations in system noise or atomic state during the k-vector reversal, the light shift cannot be completely canceled, causing measurement bias.

In this work, we study the EOM’s sideband-induced gravity measurement drift and propose a method to eliminate it. In Section 2, we use an atom gravimeter to conduct a long-term gravity measurement and find that its drift is strongly correlated with the EOM’s sideband. In Section 3, we theoretically analyze this gravity drift, construct a DLS model, and apply the measured sideband values to effectively compensate for the gravity measurement drift. In Section 4, we present a method to lock EOM’s sideband based on TA temperature modulation. This method improves the long-term stability of the sideband ratio and, therefore, improves the stability of gravity measurement.

## 2. Atom Gravimeter Measurements

**Figure 1:**
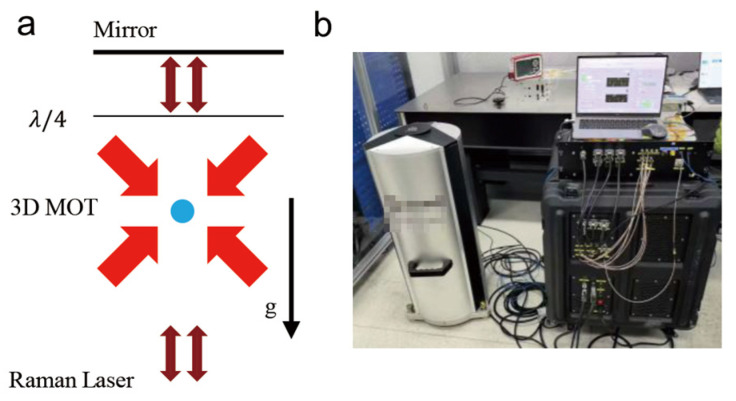
Setup of the atom gravimeter. (**a**) Schematic diagram of the atom interferometry. (**b**) Photograph of the homemade atom gravimeter.

We use a homemade atom gravimeter (as shown in Figure 1) to conduct a long-term gravity measurement [32]. The physics package is based on a titanium alloy ultra-high vacuum (UHV) system, providing an ideal environment for atomic free fall and interference. In the measurement cycle, the system loads approximately $10^{7}$ ^85^Rb atoms into a three-dimensional magneto-optical trap (3D-MOT). After polarization gradient cooling (PGC), the atomic cloud is released and falls freely in the gravitational field. During this period, the atoms are prepared into the F = 2, mF = 0 magnetically insensitive state using the Raman state selection. Then the atomic cloud undergoes a π/2−π−π/2 stimulated Raman transition pulse sequence to perform interference (Figure 1a). For this work, the selected π/2 pulse duration is 10 μs, and the free evolution time is T = 71 ms.

**Figure 2:**
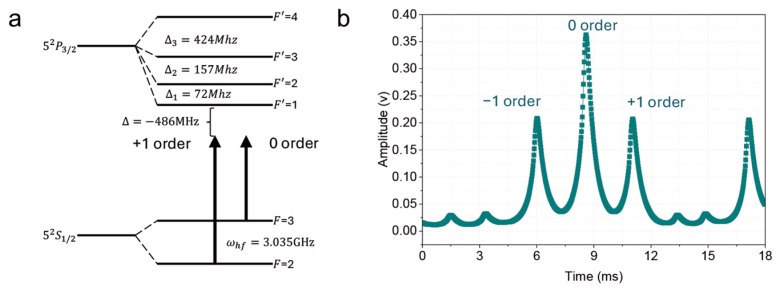
Raman laser configuration. (**a**) Energy level diagram of the ^85^Rb D_2_ transition. The ground-state hyperfine splitting frequency of ^85^Rb is $\omega_{hf}=3.035\;GHz$. (**b**) Fabry-Pérot transmission signal of the +1, 0, and −1 orders, with the −1st order locked.

The experiment employs a single-seed laser scheme. The Raman beams are generated by a phase-modulating electro-optic modulator (EOM, iXblue, Besançon, France), with its RF modulation frequency tuned to the ground state hyperfine splitting of ^85^Rb (3.035 GHz). The modulated light contains a carrier (0th order) and a pair of first-order sidebands (±1st orders), among which the carrier and the +1st order sideband contribute the major amplitude of the Raman transition (Figure 2a), while the single-photon detuning of the +1st order sideband relative to the F = 2 to F′ = 1 transition is set to red detuning Δ=−486 MHz. The laser output from the EOM is amplified by a TA and passes through an acousto-optic modulator (AOM) for pulse switching, finally being coupled into a polarization-maintaining fiber for delivery to the vacuum chamber. At the top of the vacuum chamber, the laser beam passes through a quarter-wave plate and is retro-reflected by a mirror, forming a Raman light field with a ${lin}^{+}-{lin}^{-}$ configuration.

We use a voltage-controlled Fabry-Pérot (FP) cavity to monitor the sideband ratios of the EOM. The Raman pulses are generated by switching an acousto-optic modulator (AOM), while the monitoring beam is split off before the AOM for sideband measurement. By scanning the voltage of the FP cavity, we obtain the transmission signal of the FP cavity. The scan uses a 20 ms linear voltage ramp, starting 10 ms before each Raman pulse. The transmission signal is detected by a photodetector and converted into a voltage via an I–V conversion circuit. Subsequently, we extract the magnitude of the sideband ratios through a peak detection algorithm. The FP cavity is mounted on a large heat sink, and the entire optical setup is temperature-controlled. Even after the temperature-control, the peak positions may drift. We adjust the scanning voltage range of the FP cavity to ensure we always capture ($r_{+1},\;r_{0},r_{-1}$). This allows us to consistently read the sideband ratios, although the peak positions may drift. Figure 2b shows the actual signal of the carrier and sideband data. We denote $r_{+1},r_{0},r_{-1}$ as the intensity ratios of the individual frequency components. Specifically, each ratio $r_{k}$ is defined as the optical power of the individual sideband $P_{k}$ divided by the total optical power of the Raman light $P_{total}$ ($\;r_{k}\;=\;P_{k}/P_{total}\;$, where k∈ {+1, 0, −1}). Based on the DLS theory and experimental parameters (the detailed derivation is provided in Appendix A), the ideal sideband ratios $r_{+1},r_{0},r_{-1}$ for zero light shift are 0.2707,0.4586 and  0.2707. The sum of these ratios satisfies $r_{0}+r_{1}+r_{-1}=1$. Therefore, we only discuss $r_{-1}$ and $r_{+1}$ in the following, to avoid redundancy.

**Figure 3:**
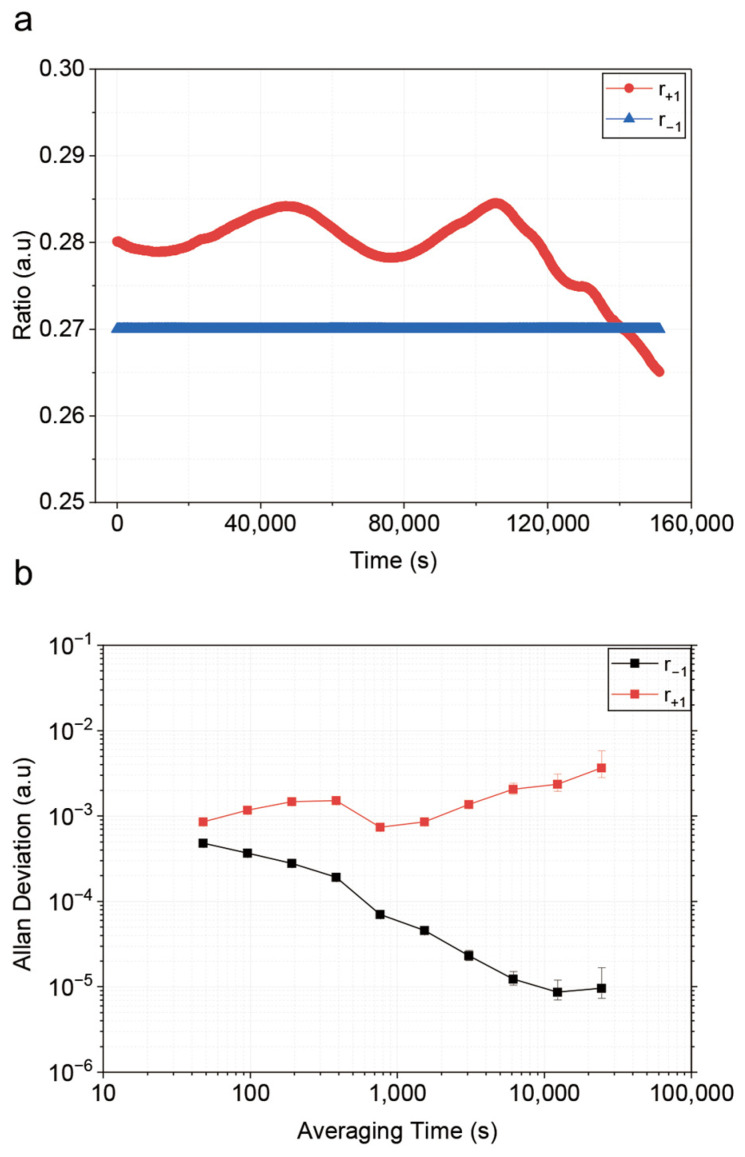
Locking of the $r_{-1}$ by feedback-controlling the power of the EOM’s microwave power. (**a**) Long-term measurement of the $r_{+1}$, $r_{-1}$ after locking. (**b**) Allan deviations of the $r_{+1}$, $r_{-1}$ after locking.

Consequently, we lock $r_{-1}$ to 0.27 by feedback-controlling the power of the EOM’s microwave driver. The feedback loop achieves a long-term stability of ${9.5\times 10}^{-6}$ for the locked sideband ratio. However, despite $r_{-1}$ being locked, a significant relative drift of $r_{+1}$ is observed, as shown in Figure 3a. The drift amplitude of $r_{+1}$ is approximately 0.02 over time. As shown in Figure 3b, the Allan deviation for $r_{+1}$ is ${3.6\times 10}^{-3}$ at a time of 24,576 s.

**Figure 4:**
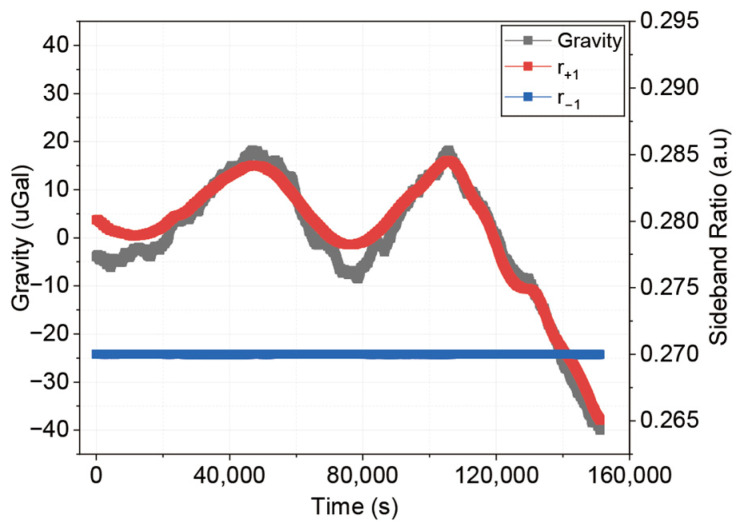
Measurement results of gravity values and $r_{\pm 1}$. Data are processed with a 120 s moving average method.

Gravity measurements are performed simultaneously with the measurement of the Raman light sideband ratios. The experiment lasted approximately 42 h (150,000 s), with a single measurement cycle time of 0.6 s. Data processing is applied to extract gravity values, including the application of k-vector reversal and the subtraction of theoretical solid tides and atmospheric pressure effects. To clearly reveal the long-term drift trends, a moving average method with a time window of 120 s is applied to both the processed gravity data and the measured sideband ratio, as shown in Figure 4. The gravity data have a standard deviation of 13.13 μGal. The scopes of measured gravity and $r_{+1}$ are strongly correlated with a correlation coefficient of $R^{2}=0.962$. Since $r_{-1}$ is already locked, the ratio drift of the $r_{+1}$ was identified as the main factor for the long-term drift in the gravity measurement.

## 3. Gravity Compensation Model

In an atom gravimeter, the measured phase Φ is composed of the gravity g-induced phase and the DLS-induced phase $\delta \Phi_{AC}$, which can be expressed as:

(1) $$\begin{array}{c} \Phi_{\pm }={\pm k}_{eff}gT^{2}+\;\delta \Phi_{AC,\pm }\;, \end{array}$$

where $k_{eff}$ is the effective Raman wave vector, g is the gravitational acceleration, T is the free evolution time. And the symbol ± represents the cases of the k-vector reversed method. The $\delta \Phi_{AC}$ arises from the differential light shifts in the Raman pulses. While a detailed theoretical derivation is provided in the Appendix A, the DLS ($\delta_{ac,i}$) for the *i*-th pulse can be expressed in terms of the actual Raman laser intensity (9.3 mW/cm^2^) and detuning as:

(2) $$\begin{array}{c} \delta_{ac,i,\pm }=2216.62r_{0}-3962.43r_{+1}+206.00r_{-1}. \end{array}$$

Here, the DLS $\delta_{ac,i,\pm }$ and the numerical coefficients are expressed in units of Hz. According to Figure 2, the ±2nd-order sidebands each account for ~4% of the total power ($P_{0}+P_{1}+P_{-1}$). As detailed in Appendix A.1, the induced DLS is only ~1.6% of the absolute DLS contributed by the primary 1st-order sideband. Therefore, they are neglected in this model. By integrating with the interferometer sensitivity function (detailed in the Appendix A), the DLS-induced phase shift takes the following form:

(3) $$\begin{array}{c} \delta \Phi_{AC,\pm }=2\left(\delta_{ac,1,\pm }-\alpha \delta_{ac,3,\pm }\right)\tau , \end{array}$$

where τ is the width of the π pulse time. The parameter α denotes the effective laser intensity differences between the first and the third Raman pulses. The DLS-induced gravity bias is then expressed as:

(4) $$\delta g^{AC,\pm }=k_{scale}\left(\delta_{ac,1,\pm }-\alpha_{\pm }\delta_{ac,3,\pm }\right)\tau ,$$

where $\delta g^{AC,\pm }$ means the gravity correction for the $k_{eff+}$ and $k_{eff-}$ situations. $k_{scale}=\frac{1}{k_{eff}T^{2}}$ is the phase to gravity scale factor. For our experiment parameters, its value is 1232 μGal/red. The final gravity correction after the k-vector reversed method is

(5) $$\delta g^{AC}=(\delta g^{AC+}+\delta g^{AC-})/2.$$

Based on the model derived from Equations (2), (4) and (5), $\delta g^{AC}$ is a function of both r and α. The r and α are physically independent. We measure the sideband ratio of $r_{+1,\pm }$, and $r_{-1,\pm }$ and gravity value for the ${\pm k}_{eff}$ interference cases. The least square method is used to obtain the optimized values of α. The fitted result is $\alpha_{-}=0.790$ and $\alpha_{+}=0.985$. The fitted α is a comprehensive result of multiple factors. It accounts for effects including the atomic cloud expansion [33] and the spatial variation in the laser field, as well as the atomic trajectory. In addition, the spatial interference of the reflected Raman beams causes the Raman transition to be location-dependent [34]. Since these contributions may counteract each other, an α parameter near one corresponds to approximately equal effective intensities for the first and third pulses.

**Figure 5:**
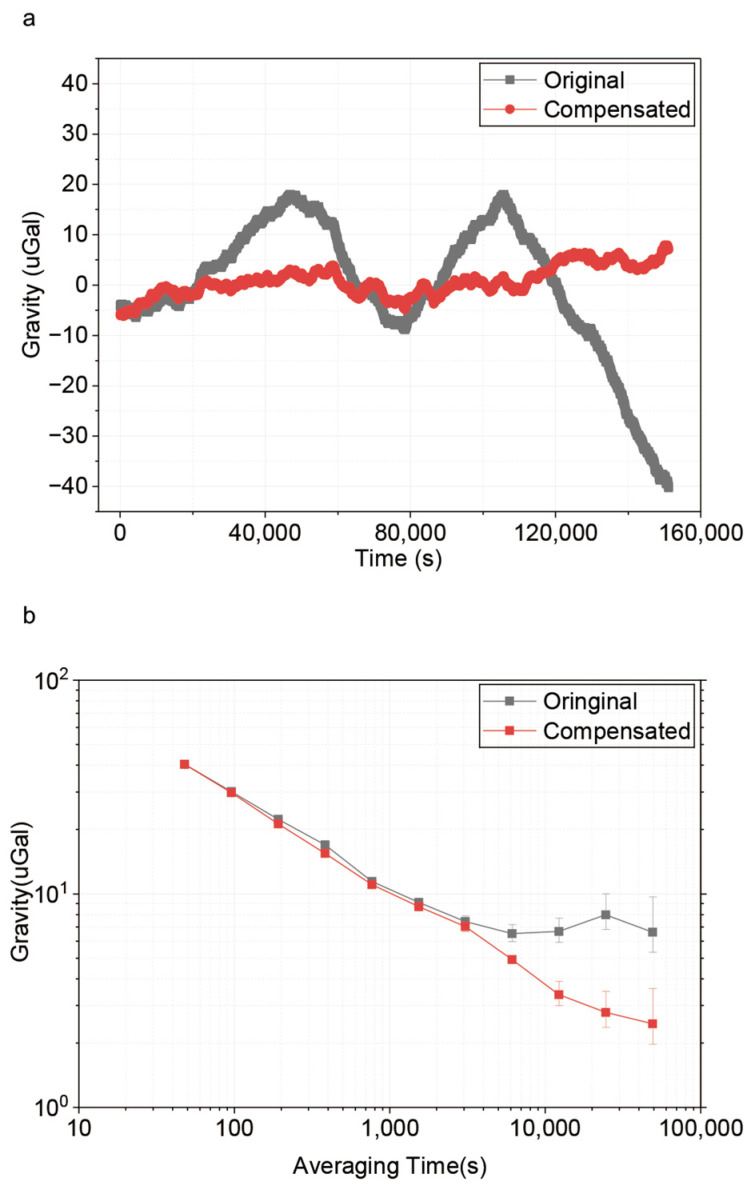
Gravity value before and after the DLS effect compensation. (**a**) Gravity values in the time domain with and without compensation. The mean value of gravity is subtracted. (**b**) Allan deviation of the gravity values with and without compensation.

Using the obtained parameters and measured sideband ratios, we perform a point-by-point compensation on the gravity data to remove its error. We call this method the differential light shift compensation (DLSC) method. Figure 5a shows that the gravity drift induced by the $r_{+1}$ variation is effectively suppressed after compensation. At an integration time of τ = 24,576 s, the Allan deviation of the gravity measurement is shown in Figure 5b. It improves from 11.1 μGal (before compensation) to 2.44 μGal and the standard deviation of the gravity measurement data (after applying moving average method) is reduced from 13.13 μGal to 2.54 μGal, which shows the efficiency of our differential light shift model.

## 4. Sideband Ratio Locking Scheme Based on TA Temperature

**Figure 6:**
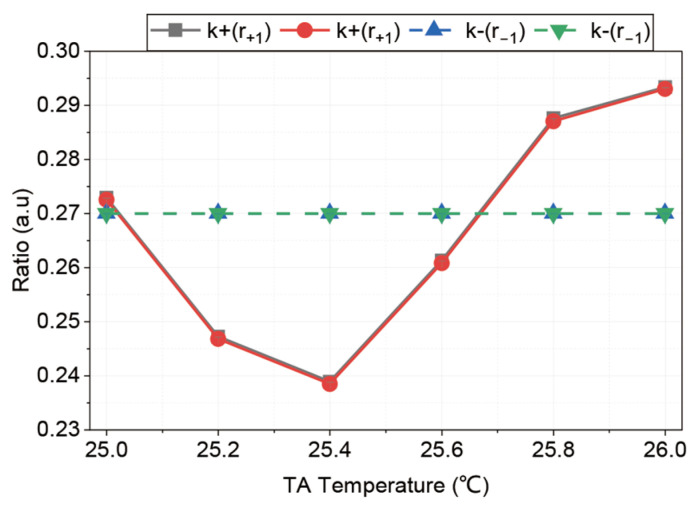
Measurement of sideband ratios with variation in the TA temperature. The solid line represents the unlocked $r_{+1}$. The dashed line represents the locked $r_{-1}$.

While the DLSC method reduces gravity drift, it relies on the parameters of the expansion coefficient α, which may vary over time and cannot be determined accurately. Therefore, active control of sideband ratios is a better way to suppress the long-term gravity drift. We varied the TA’s temperature and recorded the resulting change in sideband ratio, as shown in Figure 6. Specifically, we scanned the TA temperature while keeping the $r_{-1}$ locked via the EOM’s microwave power. Over a temperature range of 1 °C, the $r_{+1}$ changed by approximately 0.055. The possible reason for this fluctuation is the TA’s non-linear gain characteristics. Previous research [35] investigated the frequency-dependent amplification (FDA) in TAs, demonstrating that the optical gain varies with laser frequency and is sensitive to operating parameters such as saturation, temperature, and current. Based on these non-linear properties, we infer that the independent drift of the sideband ratios in our system results from mode competition among the carrier and sidebands sharing the limited gain medium.

The temperature-varied $r_{+1}$ covers the value that eliminates the differential light shift (0.27), which means that one can adjust this ratio to its proper value by adjusting the TA’s temperature. Consequently, we developed a Dual-Sideband Ratio Locking (DSRL) method that employs feedback to both the EOM’s microwave power and the TA’s temperature. This method consists of two feedback loops. The first loop uses RF control for the $r_{-1}$, by adjusting the EOM RF driving power during each Raman pulse. We lock $r_{-1}$ for each Raman pulse. The second loop adjusts the temperature control of TA to lock the $r_{+1}$. We measure the $r_{+1}$ during the middle π pulse as the input signal to feedback the TA temperature via a proportional-integral-derivative (PID) algorithm. These two loops operate independently with a sampling period of 0.6 s. Since the ±1 sidebands are coupled, we avoid control conflict by setting different integration times for the two loops. The microwave modulation loop is set to a fast response to lock the −1st order sideband, while the temperature loop feeds back on $r_{+1}$ with a slower integration time to balance ± 1 sideband ratio.

$r_{-1}$ remained locked with a long-term Allan deviation of $10^{-6}$. Compared with the single-sideband locking, the long-term Allan deviation of $r_{+1}$ improved from ${3.6\times 10}^{-3}$ to $2.8\times 10^{-5}$, corresponding to an improvement factor of 142.

We can use this fluctuation to calculate the DLS effect-induced gravity noise. According to Equations (2) and (3), the calculated result is 0.085 μGal. After applying the DSRL method, the sideband fluctuation induced by gravity noise is well below the gravity’s measurement resolution, and does not contribute to gravity measurement errors. In addition, this method is effective but does not increase optical complexity.

**Figure 7:**
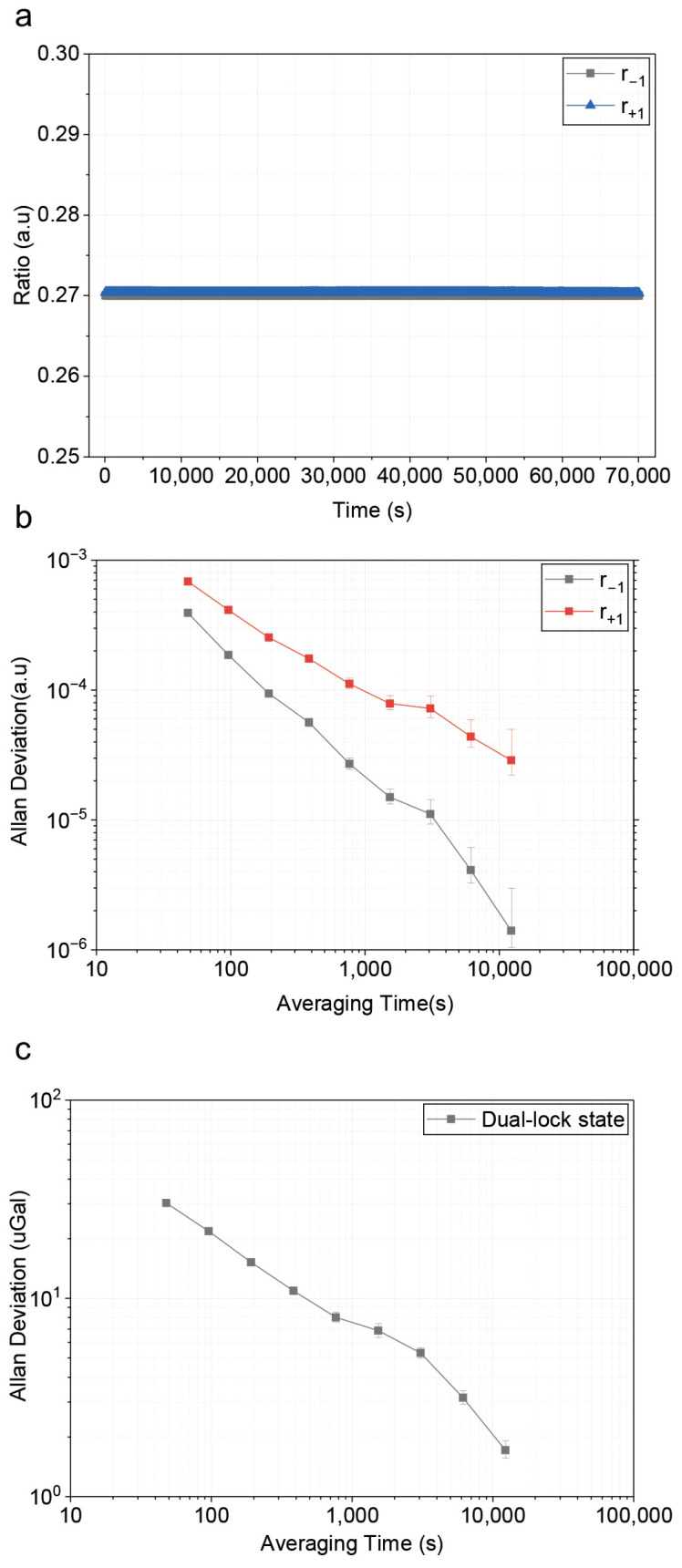
Sideband ratios and Gravity measurement result after applying the DSRL method. (**a**) Time domain measurement of $r_{\pm 1}$ and the corresponding gravity values. Data are processed with a 120 s moving average method. (**b**) Allan deviation for sideband ratios. (**c**) Allan deviation of the measured gravity.

Continuous gravity measurements were conducted for approximately 42 h (150,000 s) under the dual-locked state. The k-vector reversal method is applied and solid tides and atmospheric pressure effects are subtracted. The gravity data showed no observable drift and has a standard deviation of 0.7 μGal (after 120 s moving average filter). The Allan deviation decreases consistently with the average time and has a resolution of 1.6 μGal at an integration time of 12,288 s, as shown in Figure 7.

## 5. Conclusions

In summary, we have identified the drift of the unlocked sideband ratio (specifically, $r_{+1}$ in our system) as a major error source limiting the long-term stability of our EOM-TA-based atomic gravimeters. To actively suppress this error, we introduced a Dual-Sideband Ratio Locking method using the temperature dependence of the TA. By adding TA temperature as a feedback variable, active control of the previously drifting sideband was achieved. Experimental results show this method effectively suppresses gravity drift and enhances long-term stability without requiring complex optical modifications.

The proposed method ensures the long-term stability of the sidebands. To improve short-term stability, on one hand, it is necessary to enhance the stability of the parameters, such as the laser intensity and EOM microwave power; on the other hand, it is also possible to design a fast locking scheme within a single cycle, thereby reducing the impact of this effect on the short-term stability of gravity measurements.

The proposed DLSC method and DSRL method are not only suitable for the EOM-TA configuration but can also be extended to other Raman laser schemes. The optical phase-locked loops (OPLL) or high-frequency AOM schemes may also encounter sideband ratio drifts. The proposed method could also be applied in such schemes to improve the measurement precision of atom interferometer such as atom gravimeters, gradiometers, and gyroscopes, etc.

## Figures and Tables

**Figure A1:**
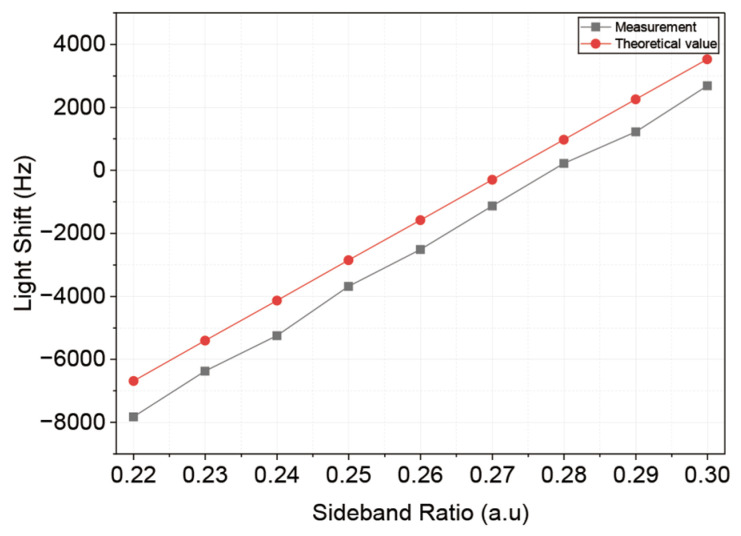
Measurement and theoretical calculation of light shift with variation in the sideband ratios.

## Data Availability

The data that support the findings of this study are available from the corresponding author upon reasonable request.

## Appendix A

### Appendix A.1. Differential Light Shift Derivation

In the far-detuning approximation, for a specific state, the AC Stark shift is the sum of contributions from all laser frequency components (k∈{−1,0,1}) and allowed transition paths ($j\in F^{\prime }$). The total shift is given by:

(A1) $$\begin{array}{c} \delta_{shift}=\underset{k,j}{\sum }\frac{{\left|\Omega_{k,j}\right|}^{2}}{4\Delta_{k,j}}, \end{array}$$

where the $\Omega_{k,j}$ is the Rabi frequency coupling the ground state to the excited state via laser component k, and $\Delta_{k,j}$ is the corresponding detuning.

For simplicity, we defined a common coefficient K, related to atomic transition strength, as:

(A2) $$\begin{array}{c} K=\frac{2d^{2}}{\hbar^{2}c\epsilon_{0}}\frac{1}{2\pi }, \end{array}$$

where d is the dipole matrix element, ℏ is the Planck constant, c is the vacuum light speed and $\epsilon_{0}$ is vacuum permittivity.

We consider the total laser intensity $I_{total}$, and sideband ratio $r_{k}$. For the specific case of ^85^Rb, substituting Equation (A2) into Equation (A1) yields the AC Stark shift for clock states $\left.|F=2,m_{F}=0\right\rangle$ and $\left.|F=3,m_{F}=0\right\rangle$:

(A3) $$\begin{array}{c} \delta_{ac,F=2}=\frac{{KI}_{total}}{4}\left(\begin{array}{c} r_{+1}\left[\frac{1}{20\Delta }+\frac{7}{36\left(\Delta +\Delta_{1}-\Delta_{2}\right)}+\frac{4}{45\left(\Delta +\Delta_{1}-\Delta_{3}\right)}\right]+\\ r_{0}\left[\begin{array}{c} \frac{1}{20\left(\Delta -\omega_{HF}\right)}+\frac{7}{36\left(\Delta +\Delta_{1}-\Delta_{2}-\omega_{HF}\right)}\\ +\frac{4}{45\left(\Delta +\Delta_{1}-\Delta_{3}-\omega_{HF}\right)} \end{array}\right]+\\ r_{-1}\left[\begin{array}{c} \frac{1}{20\left(\Delta -2\omega_{HF}\right)}+\frac{7}{36\left(\Delta +\Delta_{1}-\Delta_{2}-2\omega_{HF}\right)}\\ +\frac{4}{45\left(\Delta +\Delta_{1}-\Delta_{3}-2\omega_{HF}\right)} \end{array}\right] \end{array}\right), \end{array}$$

(A4) $$\begin{array}{c} \;\;\delta_{ac,F=3}=\frac{KI_{total}}{4}\left(\begin{array}{c} r_{+1}\left[\begin{array}{c} \frac{1}{63\left(\Delta +\Delta_{1}-\Delta_{2}+\omega_{HF}\right)}+\frac{5}{36\left(\Delta +\Delta_{1}-\Delta_{3}+\omega_{HF}\right)}\\ +\frac{5}{28\left(\Delta +\Delta_{1}-\Delta_{4}+\omega_{HF}\right)} \end{array}\right]\\ +r_{0}\left[\begin{array}{c} \frac{1}{63\left(\Delta +\Delta_{1}-\Delta_{2}\right)}+\frac{5}{36\left(\Delta +\Delta_{1}-\Delta_{3}\right)}\\ +\frac{5}{28\left(\Delta +\Delta_{1}-\Delta_{4}\right)} \end{array}\right]+\\ r_{-1}\left[\begin{array}{c} \frac{1}{63\left(\Delta +\Delta_{1}-\Delta_{2}-\omega_{HF}\right)}+\frac{5}{36\left(\Delta +\Delta_{1}-\Delta_{3}-\omega_{HF}\right)}\\ +\frac{5}{28\left(\Delta +\Delta_{1}-\Delta_{4}-\omega_{HF}\right)} \end{array}\right] \end{array}\right), \end{array}$$

Here, Δ represents the single-photon detuning, $\Delta_{i}$ denotes the energy separation between the excited hyperfine level $F^{\prime }=j$ and the $5^{2}P_{3/2}$ fine-structure level. $\omega_{HF}$ is the ground state hyperfine splitting and $I_{total}$ represents the total Raman laser intensity.

Finally, we define the total DLS $\delta_{ac,i}$ as the difference between the shifts in the two ground hyperfine states:

(A5) $$\begin{array}{c} \delta_{ac,i}=\delta_{ac,F=2}-\delta_{ac,F=3}\;, \end{array}$$

By substituting the atomic parameters [36] and calculating the numerical values, the differential light shift simplifies to a function of the total intensity $I_{total}$ and the sideband ratios:

(A6) $$\begin{array}{c} \delta_{ac}=I_{total}\left(238.35r_{0}-426.07r_{+1}+22.15r_{-1}\right), \end{array}$$

This general expression corresponds to the specific shift $\delta_{ac}$ for the i-th pulse used in Equation (2), where the total intensity $I_{toal}$ is replaced by the specific pulse intensity $I_{i}$. Based on Equation (A6), DLS can be theoretically eliminated by appropriately tuning the sideband ratios. Solving for the condition $\Delta \delta_{ac}=0$, we obtain the optimal sideband ratio where $r_{+1}=r_{-1}=0.2707.$

Based on Equation (A1), we extended the DLS formula to explicitly include ±2nd-order sidebands. The generalized expression for the DLS becomes:

(A7) $$\begin{array}{c} \delta_{ac}=I_{total}\left(238.35r_{0}-426.07r_{+1}+22.15r_{-1}+39.44r_{+2}+8.31r_{-2}\right), \end{array}$$

As shown in Figure 2, the typical +2nd and −2nd order sidebands relative to the total power ($P_{0}+P_{1}+P_{-1}$) are 0.032 and 0.03, respectively. Due to their larger effective single-photon detuning, the combined DLS magnitude contributed by the 2nd-order sidebands is approximately 1.6% of the absolute magnitude contributed by the primary 1st-order sidebands. Their effect becomes a negligible gravity measurement bias.

### Appendix A.2. Effective Rabi Frequency

The two-photon effective Rabi frequency $\Omega_{eff}$ driving the Raman transition is given by the sum over intermediate states j and allowed laser pairs:

(A8) $$\begin{array}{c} \Omega_{eff}=\underset{k,k^{\prime },j}{\sum }\frac{\Omega_{k,j}\cdot (\Omega_{k^{\prime },j}{)}^{*}}{2\Delta_{k,j}}, \end{array}$$

where the indices k and k′ denote the laser frequency that satisfy the resonance conditions. $\Delta_{k,j}$ is the effective detuning from the intermediate state j, which is determined by the single-photon detuning Δ and the energy separation $\Delta_{j}$ (consistent with A.1). Using the previously defined coefficient K and the sideband ratio $r_{k}$, the explicit expression for our system is:

(A9) $$\begin{array}{c} \Omega_{eff}=\frac{KI_{total}}{2}\left(\begin{array}{c} \sqrt{r_{+1}r_{0}}\left[\frac{\frac{2}{18}}{\Delta +\Delta_{1}-\Delta_{2}}+\frac{\frac{2}{9}}{\Delta +\Delta_{1}-\Delta_{3}}\right]+\\ \sqrt{r_{0}r_{-1}}\left[\frac{\frac{2}{18}}{\Delta +\Delta_{1}-\Delta_{2}-\omega_{HF}}+\frac{\frac{2}{9}}{\Delta +\Delta_{1}-\Delta_{3}-\omega_{HF}}\right] \end{array}\right), \end{array}$$

Substituting the parameters yields the numerical approximation:

(A10) $$\begin{array}{c} \Omega_{eff}=I_{total}\left(660.891\sqrt{r_{0}r_{+1}}+102.263\sqrt{r_{0}r_{-1}}\right), \end{array}$$

According to Equation (A9) and the actual π/2 pulse time of 10 μs, the effective Raman laser intensity is calculated as 9.3 mW/cm^2^.

### Appendix A.3. Phase Shift Induced by DLS

In atom interferometry, noise is transferred to the final phase measurement through the interferometer sensitivity function. The total phase error $\delta \Phi_{AC}$ caused by the light shift can be expressed by the time-domain integral of the sensitivity function g(t) and the instantaneous differential light shift $\delta_{ac,i}(t)$:

(A11) $$\begin{array}{c} \delta \Phi_{AC}=\int g\left(t\right)\delta_{ac,i}\left(t\right)\mathrm{dt}, \end{array}$$

where $\delta_{ac,i}(t)$ represents the differential light shift in the i-th Raman pulse. For a π/2−π−π/2 pulse sequence, where τ represents the duration of the central π-pulse (and τ/2 represents the duration of the π/2-pulses), and T represents the free evolution time, the sensitivity function g(t) [37] is explicitly defined as:

(A12) $$\begin{array}{c} \;\;\;\;\;\;\;\;\;g\left(t\right)=\left\{\begin{array}{cc} -\sin\left(\frac{\pi t}{\tau }\right) & 0\leq t<\frac{\tau }{2}\\ -1 & \frac{\tau }{2}\leq t<T+\frac{\tau }{2}\\ -\cos\left(\frac{\pi }{\tau }\left(t-T-\frac{\tau }{2}\right)\right) & T+\frac{\tau }{2}\leq t<T+\frac{3\tau }{2}\\ 1 & T+\frac{3\tau }{2}\leq t<2T+\frac{3\tau }{2}\\ \cos\left(\frac{\pi }{\tau }\left(t-2T-\frac{3\tau }{2}\right)\right) & 2T+\frac{3\tau }{2}\leq t\leq 2T+2\tau \\ 0 & \mathrm{otherwise} \end{array}\right. \end{array}$$

We assume the laser intensity is constant within the short pulse duration τ. Substituting Equation (A6) into Equation (A10) yields the total phase shift.

(A13) $$\begin{array}{c} \delta \Phi_{AC}=2\left(\delta_{ac,1}-\delta_{ac,3}\right)\tau \end{array}$$

However, $\delta_{ac,i}(t)$ depends on the spatial overlap between the atomic cloud and the Raman laser. As the atomic cloud falls and expands, the atoms experience varying effective light intensities at each pulse timing, thereby leading to a difference between $\delta_{ac,1}(t)$ and $\delta_{ac,3}(t)$, resulting in DLS phase $\delta \Phi_{AC}$ given by Equation (3).

### Appendix A.4. Sideband Ratio Modulation Experiment

We conducted a sideband ratio modulation experiment by varying the sideband ratios $r_{\pm 1}$ and measuring the changes in light shift. Figure A1 shows the relationship between the measured light shift and the sideband ratios.

As shown in Figure A1, the measured light shift varies with the sideband ratio. We calculate a theoretical value of light shift with the same Raman laser intensity using Equation (A7). In the experiment, the DLS is zero and becomes completely independent of the total laser intensity at the sideband ratio $r_{+1}=0.27$. This result is consistent with our theoretical optimal ratio of 0.2707 derived in Equation (A6). Furthermore, the measured light shift follows the same trend as the theoretical prediction as the sideband ratio varies, providing direct experimental evidence that the gravity drift observed in our system is indeed caused by the differential light shift.
